# Optimization of Culture Medium for the Production of an Exopolysaccharide (p-CY02) with Cryoprotective Activity by *Pseudoalteromonas* sp. RosPo-2 from the Antarctic Sea

**DOI:** 10.4014/jmb.2402.02037

**Published:** 2024-03-19

**Authors:** Pilsung Kang, Sung Jin Kim, Ha Ju Park, Il Chan Kim, Se Jong Han, Joung Han Yim

**Affiliations:** 1 Development of Biomaterials from Polar Region, Korea Polar Research Institute, Incheon 21990, Republic of Korea; 2 CRYOTECH Inc., Busan 46744, Republic of Korea

**Keywords:** Cryoprotective activity, exopolysaccharide, the Antarctic Sea, response surface methodology

## Abstract

When cells are exposed to freezing temperatures, high concentrations of cryoprotective agents (CPA) prevent ice crystal formation, thus enhancing cell survival. However, high concentrations of CPAs can also cause cell toxicity. Exopolysaccharides (EPSs) from polar marine environments exhibit lower toxicity and display effects similar to traditional CPA. In this study, we sought to address these issues by i) selecting strains that produce EPS with novel cryoprotective activity, and ii) optimizing culture conditions for EPS production. Sixty-six bacteria producing mucous substances were isolated from the Ross Sea (Antarctic Ocean) using solid marine agar plates. Among them, *Pseudoalteromonas* sp. RosPo-2 was ultimately selected based on the rheological properties of the produced EPS (p-CY02). Cryoprotective activity experiments demonstrated that p-CY02 exhibited significantly cryoprotective activity at a concentration of 0.8% (w/v) on mammalian cells (HaCaT). This activity was further improved when combined with various concentrations of dimethyl sulfoxide (DMSO) compared to using DMSO alone. Moreover, the survival rate of HaCaT cells treated with 5% (v/v) DMSO and 0.8% (w/v) p-CY02 was measured at 87.9 ± 2.8% after freezing treatment. This suggests that p-CY02 may be developed as a more effective, less toxic, and novel non-permeating CPA. To enhance the production of EPS with cryoprotective activity, Response Surface Methodology (RSM) was implemented, resulting in a 1.64-fold increase in production of EPS with cryoprotective activity.

## Introduction

To prevent cell death during freezing, cryoprotective agents (CPAs), such as dimethyl sulfoxide (DMSO) and glycerol, can be added to prevent the formation of ice crystals within cells [1]. CPAs induce cell dehydration to suppress the formation of ice crystals and lower the freezing point, promoting vitrification. This approach helps protect cells in freezing environments [2]. To achieve these effects, high concentrations of CPAs are commonly employed, yet elevated concentrations of CPAs cause significant toxicity to cells [3]. Therefore, to attain optimal vitrification conditions, it is necessary to explore the development of new CPAs or find a balance between concentration and toxicity.

When exposed to subzero temperatures, biological organisms experience the formation of ice crystals within their cells. The structural damage to cells induced by ice crystals leads to excessive water leakage, protein denaturation, metabolic disruptions, and the accumulation of reactive oxygen species (ROS), ultimately resulting in cell death [4]. In polar winter, sea ice forms on the sea surface and provides extreme habitat conditions for many organisms, including a broad range of bacterial, fungal, algal, protozoan and metazoan species [5, 6].

These organisms can survive in environments of extreme temperature, salinity, and osmotic pressure conditions [7, 8], because they generate abundant quantities of EPSs stored as thick gels that envelop them [9-13]. The mechanism for this survival strategy is based on the fact that high EPS concentration has appreciable effects on system freezing point depression [14, 15]. Additionally, the viscosity of EPS is increased by the hydrogen bonding between multiple hydrophilic side chains at low temperature, and this increased viscosity inhibits ice crystal formation [15, 16].

EPSs produced by microorganisms isolated from cold marine environments have been extensively studied for the development of cryoprotective EPSs [8, 12, 17, 18]. Among the various advantages for industry of EPSs produced by bacterial cells from cold marine environments include low cost, low toxicity, high selectivity, and specific activity at extreme temperatures and biodegradability [19, 20]. EPSs derived from plants and animals are challenging to extract and purify from organic organisms. Moreover, seasonal variations impact the yield and production costs associated with plant- and animal-derived EPS, making large-scale production and utilization difficult [21]. EPSs produced by bacteria, however, have a short production cycle, which enables high production efficiency throughout the fermentation process. In addition, the easy purification process of microbial EPS addresses issues associated with plant and animal-derived EPS, making them advantageous for industrial application [21-23]. The traditional method of analyzing responses by changing one independent variable at a time is time-consuming and cost-intensive, and cannot be used to determine the interactions between independent variables [24]. The use of Response Surface Methodology (RSM), on the other hand, enables simultaneous investigation of the interactions among various independent variables [25], while also optimizing the process by minimizing operational costs saving time [26]. Furthermore, this optimization tool can be widely applied to various aspects, such as the composition of the medium and fermentation conditions for EPSs, which further helps to cut costs and duration [27-30].

As such, in this study we sought to isolate bacteria that produce cryoprotective EPS and investigate the effects of the medium composition on the production yield of EPSs using RSM with the selected strains.

## Materials and Methods

### Screening of Exopolysaccharide-Producing Bacteria

Marine samples of various types (seawater, sediment, invertebrates, and benthic animals) were collected at different sites in the Ross Sea (location: 73.0-77.0° S, 167° E-163° W) during the Antarctic Sea expedition cruise of the Korean icebreaker Araon (December-February, 2012-2013). Seawater samples were collected at various depths using a Niskin bottle system equipped with a Conductivity-Temperature-Depth (CDT) sensor [31]. Bacteria present in the seawater were concentrated using a 0.2 μm filter. The concentrated bacteria were then diluted in sterilized seawater. Deep-sea sediment samples were collected using a box-core sampler [32], and the sediment was suspended in sterilized seawater. Using a dredge, we gathered invertebrates and benthic animals from the sea floor [33]. After collection, the samples were washed five times with sterilized seawater. Following this, the samples were ground using a homogenizer and then suspended in sterilized seawater. The preprocessed marine samples were cultured in marine agar plates (BD Difco., USA) at 15°C for 7 days for primary screening of EPS-producing strains. These strains were screened according to their colony stickiness and ropiness characteristics [34]. The isolated strains were inoculated into Marine Broth (MB; DB Difco) and cultured at 15°C and 120 rpm for one day. After adding glycerol to the culture medium to achieve a 20% glycerol solution, each strain was stored at -80°C until the next experiment. Further screening of the EPS-producing strains was performed according to the methods of Kim *et al*. [35].

### Cell Culture Conditions and EPS Purification

For the seed culture, each of the glycerol stock of 66 strains was inoculated into MB medium and cultured at 15°C and 120 rpm until reaching an optical density at 600 nm (OD_600_ nm), value of 3. The seed was inoculated at a concentration of 5% (v/v) in 25 ml of MB medium containing 2.0% (w/v) glucose (MBG) and was incubated at 15°C and 120 rpm for 72 h (main culture). The sample cultured in 25 ml of MBG medium was separated into cells and culture broth by centrifugation (at 12,000 ×*g* and 25°C for 10 min). The cells were washed three times with deionized water (DW) to recover the EPSs attached to the cell surface.

The culture medium and DW used for washing were mixed, and the EPSs were extracted from the mixture via the addition of two volumes of chilled ethanol (EtOH). The EPSs floating on the solution were harvested and then centrifuged to remove the supernatant from the EPSs. The precipitated EPSs were then dissolved in 25 ml of DW. This EPS solution was dialyzed for approximately 16 h using 10 kDa dialysis tubing (Thermo Fisher Scientific, USA). The dialyzed EPS solution was lyophilized (crude EPSs), after which the dry weight was measured.

Purification prior to the cryoprotective activity assay was performed according to the methods of Kim and Yim [12]. Crude EPSs were dissolved in DW and treated for 1 h with protease (500 units/L) at 37°C. Then, the mixture was centrifuged at 12,000 ×*g* at 25°C for 10 min. The deproteinated crude EPS solution was reprecipitated via the addition of a 3.0% (w/v) solution of cetylpyridinium chloride (CPC; Sigma, USA). After centrifugation (at 12,000 ×*g* and 25°C for 10 min), the supernatant was removed. The precipitated EPS-CPC complex was redissolved in 10.0% (w/v) NaCl. EPSs were separated from the EPS-CPC complex solution with two volumes of chilled EtOH. EPSs were redissolved in DW, dialyzed against 100 kDa Viva-Flow (Sartorius, Germany) and DW until the conductivity of the wastewater reached zero, after which the mixture was lyophilized.

### Rheological Properties

The rheological properties of the various strain-derived EPSs were measured by the method described in Kim *et al*. [35]. The rheological behavior, including shear rate vs. shear stress, was measured at 0.3% (w/v) EPSs. This approach facilitated the comparison and verification of the relative properties of each EPS. The viscosity of the solutions was measured using an LTV rotational spindle viscometer (DV-II, Brookfield, USA) equipped with a spindle (No. S18).

### Cryoprotective Activity of Strains from the Antarctic Sea

To measure the cryoprotective activity of the EPS produced by RosPo-1 and RosPo-2 strains, we employed the HaCaT cell line, which is sensitive to freeze-induced damage [36] and is widely used in tissue engineering research [37]. HaCaT cells (5×10^5^ cells/ml/vial) were suspended in Dulbecco’s modified Eagle medium (DMEM) supplemented with each EPS at different concentrations [0.1~1.0% (w/v)]. These mixtures were frozen at -80°C for 1 h and thawed quickly in 37°C waters for 5 min. The cell viability based on the cryoprotective properties of each EPS was measured according to the manufacturer’s instructions for the CytoTox-ONE™ Homogeneous Membrane Integrity Assay Kit (Promega, USA). The percentage of cell viability was calculated using the following formula:


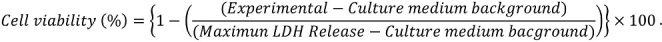


Then, HaCaT cells (5×10^5^ cells/ml/vial) were suspended in DMEM supplemented with different concentrations [1.0~5.0% (v/v)] of DMSO with 0.8% (w/v) EPS (p-CY02) produced by RosPo-2 strain, or different concentrations [1.0~5.0% (v/v)] of DMSO alone. The freeze–thaw cycle and cryoprotective property determination methods used were the same as those described in the preceding paragraph.

### Identification

The selected RosPo-2 strain was identified using 16S rRNA gene sequencing, and phylogenetic analyses were carried out according to the methods described by Baek *et al*. [38]. The 16S rRNA gene sequence (1,399 bp) of the RosPo-2 strain was compared with sequences available in the EzTaxon database (www.eztaxon.org) [39]. The 16S rRNA gene sequence was aligned with those of its closest relatives using the RDP II online aligner (http://rdp.cme.msu.edu/index.jsp) [40]. Phylogenetic trees were reconstructed using the neighbor-joining method [41] based on the Jukes–Cantor distance [42], the maximum parsimony method [43], and the maximum likelihood method [44] via the MEGA 6 program [45]. The robustness of the phylogenetic trees generated by the three tree-making algorithms was confirmed by bootstrap analyses based on 1000 random sequence re-samplings.

### Plackett–Burman Design

The Plackett–Burman design [46] was used to identify the essential components that significantly influence the production of p-CY02. In a preliminary experiment, we screened for the type and concentration of organic source (1.5% (w/v) fructose and 2.0% (w/v) yeast extract) for the optimal production of EPSs by RosPo-2 (data not shown). Based on the design, 14 nutrient components (C_6_H_5_FeO_7_, NaCl, MgCl_2_∙ 6H_2_O, Na_2_SO_4_, CaCl_2_∙2H_2_O, KCl, NaHCO_3_, KBr, SrCl_2_, H_3_BO_3_, Na_2_SiO_3_, NaF, NH_4_NO_3_ and Na_2_HPO_4_) of MB were examined at two concentration levels [low level (-1) and high level (+1)] to identify components with negative effects, as shown in Tables S1 and S2 (Plackett‒Burman design for elimination). To determine the factors that significantly influence EPS production, the selected components (C_6_H_5_FeO_7_, NaCl, MgCl_2_, Na_2_SO_4_, CaCl_2_, KCl, KBr, H_3_BO_3_, NH_4_NO_3_, and Na_2_HPO_4_) were again examined using the Plackett–Burman design method (Tables S4 and S5).

In the first-order model, Y= β_0_ + Σβ_i_X_i_ , where Y is the predicted response (p-CY02 production), β_0_ is the model intercept, β_i_ is the linear coefficient, and X_i_ is the level of the independent variable, the model does not describe interactions among factors (nutrient components) and was used only to screen and evaluate important factors influencing the response. The statistical software Minitab (v. 14.1; Minitab, Inc., USA) was used for the experimental design and for regression analysis of the data obtained. A *p* value of less than 0.05 was considered to indicate statistical significance.

### Central Composite Design

To optimize the concentrations of the nutrient components previously selected through the experiment using the Plackett–Burman design, a central composite design was applied [47]. The variables and experimental conditions for the central composite design are shown in Tables 1 and 2.

For prediction of the optimal concentrations, a second-order polynomial model was designed to describe the relationship between the independent variables (nutrient components) and the response: Y = β_0_ + Σβ_i_X_i_ + Σβ_ij_X_i_X_j_+ Σβ_ii_X_j_^2^, where Y is the predicted response (p-CY02 production); and β_0_, β_i_, β_ij_ and β_ii_ are the constant and regression coefficients of the model, with X_i_ and X_j_ representing the independent nutrient components. The statistical software Minitab (v. 14.1; Minitab, Inc.) was used for the experimental design and for regression analysis of the data obtained. A *p* value of less than 0.05 was considered to indicate statistical significance.

## Results

### Isolation and Selection

**Fig. 1:**
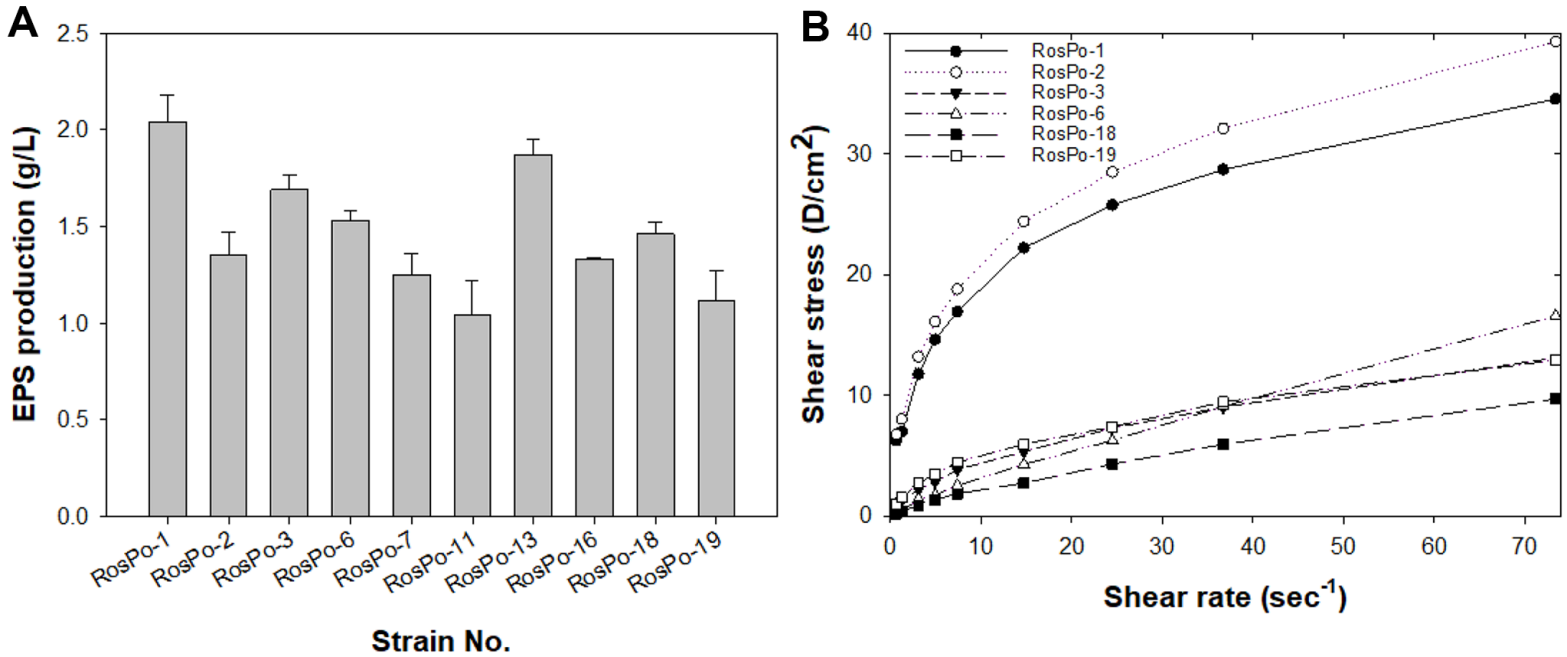
The assessment of EPS production and viscosity for each strain. (**A**) EPS-producing microorganisms, 2^nd^ selection. The strains were cultured for 3 days at 15°C in MBG medium. EPS production was estimated via its dry weight. The values shown are the means ± SDs from three experimental repeats [35; Fig. S1]. (**B**) Comparisons of the shear stress and shear rate on 0.3% (w/v) EPS. Note: RosPo-7, -11, -13, and -16 showed minimal changes in shear stress and shear rate; therefore, they are not presented on this graph.

**Fig. 2:**
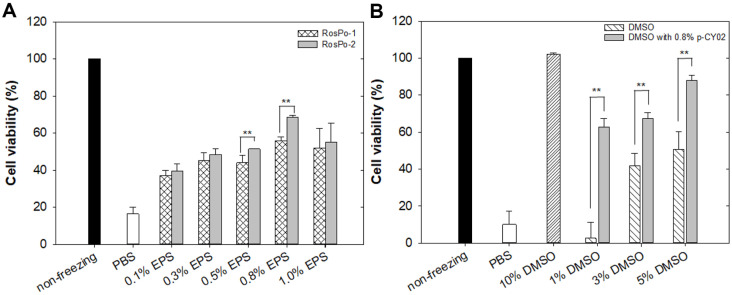
Comparison of cryoprotective activity in HaCaT cells for EPS produced by RosPo-1 and RosPo-2, and various concentrations of DMSO containing 0.8% p-CY02. (**A**) HaCaT cells were suspended in various concentrations of EPS produced by RosPo-1 and RosPo-2 strain, frozen at -80°C for 1 h, and subsequently thawed at 37°C for 5 min. (**B**) HaCaT cells were frozen in the presence of DMSO alone or in combination with 0.8% (w/v) p-CY02 at -80°C for 1 h and subsequently thawed at 37°C for 5 min. “Non-freezing” means that there are no cell death experiments because there is no freeze process in the medium. Non-freezing: HaCaT cell in DMEM, PBS: HaCaT cell in PBS and 10% DMSO: HaCaT cell in 10% DMSO. Error bars represent the standard deviation of the mean values of three independent experiments. Damaged cell ratios were determined by an LDH cytotoxicity fluorometric assay. The mean of the sharp symbols represents the control group, and the mean of the asterisks indicates statistical comparisons between the control group and treatment groups. Significance: ** *p* < 0.05.

In total, 66 strains that produced mucous substances were isolated from marine agar plate using the loop touch test, and EPS-producing strains were screened according to their colony stickiness and ropiness characteristics (1^st^ selection; data not shown) [34, 48]. Subsequently, the first selected microorganisms were cultured in MBG medium, and crude EPSs were extracted using chilled EtOH. Among the first selected microorganisms, 10 species were reselected based on having a crude EPS dry weight equal to or exceeding 1 g/l (2^nd^ selection; Fig. 1A). The EPSs produced from the 2^nd^ group of selected microorganisms were purified, after which the viscosity of the purified EPSs was measured (Fig. 1B). A polysaccharide solution exhibits changes in viscosity with respect to shear rate, and the curve shows the characteristic shape of a logarithmic decrement curve; furthermore, the curve representing shear stress increases with increasing shear rate, displaying varied shapes [49]. Differences in shear stress were evident depending on the microbial-derived EPS. This result indicated that the production of EPSs with varying viscosities depended on the strain (Fig. 1B). These features, typically observed in hyperbolic polysaccharide solutions, suggest that the majority of the EPSs analyzed in this study exhibited the characteristics of a soluble gum with pseudoplastic properties, which are typical of non-Newtonian fluids. Each EPS produced by strains RosPo-7, -11, -13, and -16 exhibited minimal increases in shear stress despite the increase in shear rate (data not shown). Conversely, EPS produced by strains RosPo-1, -2, -3, -6, -18, and -19 exhibited shear rate-dependent increases in shear stress, with EPS produced by RosPo-1 and -2 exhibiting a notably sharp increase. For final selection, the cryoprotective activity of adherent cells, including HaCaT cells, was measured using EPS produced by RosPo-1 and RosPo-2 strain (Fig. 2A). HaCaT cells were treated with various concentrations [0.1~1.0% (w/v)] of each EPS. A comparison of the survival rates between the control [non-freezing, phosphate-buffered saline (PBS)] and EPS treatment groups revealed that both EPS exhibited a concentration-dependent increase in the survival rate of the HaCaT cells. In the case of EPS produced by RosPo-1 strain, the highest survival rate was observed at 0.8% EPS (55.9 ± 2.0%), while 1.0% EPS exhibited a lower survival rate (51.9 ± 10.6%) compared to 0.8% EPS produced by RosPo-1 strain. Moreover, in the case of EPS produced by RosPo-2 strain, the highest survival rate was observed at 0.8% EPS (68.7 ± 0.9%), while 1.0% EPS exhibited a lower survival rate (55.1± 10.4%) compared to 0.8% EPS produced by RosPo-2 strain (Fig. 2A). The comparative analysis of the cryoprotective activity of EPS produced by RosPo-1 and RosPo-2 strain revealed that EPS produced by RosPo-2 strain exhibited a higher antifreeze effect in HaCaT cell compared to the EPS produced by RosPo-1 strain. Although the production of EPS was found to be higher in RosPo-1 strain compared to RosPo-2 strain, RosPo-2 strain was selected for further study due to its cryoprotective properties for HaCaT cells. The EPS produced by RosPo-2 strain was named as “p-CY02.”

To investigate whether p-CY02 can reduce the concentration of commonly used DMSO, HaCaT cells were treated with a combination of 0.8% p-CY02 and various concentrations (1.0~5.0%, w/v) of DMSO, or with different concentrations of DMSO alone, after which the survival rate was measured. The survival rate increased in a concentration-dependent manner in the group treated with DMSO alone. Additionally, when comparing the group treated with DMSO alone and the group treated with a combination of p-CY02 and DMSO, there was a concentration-dependent increase in the survival rate, which was similar to that of the DMSO-only group. The results indicated that the survival rates of the DMSO-treated group and the group treated with a combination of p-CY02 and DMSO increased with increasing concentrations of DMSO. Furthermore, when comparing the group treated with a combination of p-CY02 and DMSO to the group treated with DMSO alone, there was a significant increase in the survival rate: the survival rate was 23.6, 1.6, and 1.7 times greater in the 1%, 3%, and 5% (v/v) DMSO plus p-CY02 groups, respectively, than in the group treated with DMSO alone. Moreover, compared with those of HaCaT cells treated with 10% (v/v) DMSO, the viability of HaCaT cells treated with 5% DMSO and 0.8%p-CY02 was approximately 14.3% lower, exhibiting a survival rate of 87.9 ± 2.8% (Fig. 2B).

### Identification of the RosPo-2 Strain

**Fig. 3:**
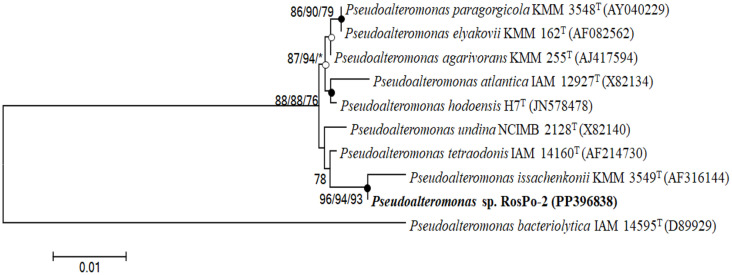
Maximum-likelihood phylogenetic tree based on 16S rDNA gene sequences showing the relationship between the *Pseudomonas* sp. RosPo-2 (PP396838) and other *Pseudomonas* species. Bootstrap values (>60%) based on 1000 replicates are shown on corresponding branches (maximum likelihood method / neighbor-joining method / maximum-parsimony method). Asterisks denote bootstrap values less than 60%. Filled circles indicate conserved nodes in maximum likelihood method, neighbor-joining method and maximum-parsimony method threes with bootstrap values more than 70%. *Pseudoalteromonas bacteriolytica* IAM14595^T^ (D89929) was used as an outgroup. Scale bar, 0.01 substitutions per nucleotide position.

A comparison of the 16S rRNA gene sequence revealed that the selected RosPo-2 strain (1399 bp) was closely related to *Pseudoalteromonas tetraodonis* IAM 14160^T^ (Accession number: AF214730; 99.86% 16S rRNA gene sequence similarity), *Pseudoalteromonas issachenkonii* KMM 3549^T^ (AF316144; 99.85%), *Pseudoalteromonas undina* NCIMB 2128^T^ (X82140; 99.49%) and *Pseudoalteromonas espejiana* NCIMB 2127^T^ (X82143; 99.49%). In all the phylogenetic trees generated using the 16S rRNA gene sequence in this study, the RosPo-2 and *P. issachenkonii* KMM3549^T^ (AF316144) with valid published names constituted a robust clade (Fig. 3). This aspect was shown in three algorithms; based on the bootstrap support values, robust was demonstrated. According to these results, the RosPo-2 strain was judged to be influenced by *Pseudoalteromonas* sp. RosPo-2 (PP396838).

### Plackett–Burman Design

To eliminate nutrient components that negatively affect p-CY02 production, 14 nutrient components were prepared at two concentrations (-1, +1) (Table S1). The design table and p-CY02 production data are shown in Table S2. The polynomial model describing the correlation between the fourteen components and the predicted p-CY02 production is presented as follows:


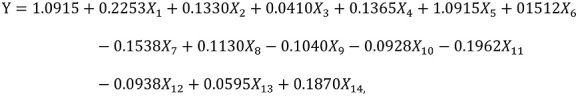


where Y is the predicted response (p-CY02 production) and X1-X_14_ are the coded values of C_6_H_5_FeO_7_, NaCl, MgCl_2_, Na_2_SO_4_, CaCl_2_, KCl, NaHCO_3_, KBr, SrCl_2_, H_3_BO_3_, Na_2_SiO_3_, NaF, NH_4_NO_3_, and Na_2_HPO_4_, respectively. Consequently, Table S3 shows the effect, t statistics, and *p* value for each nutrient component. The *p* values for NH_4_NO_3_, MgCl_2_ and H_3_BO_3_ were > 0.05, indicating that these components are not significant factors affecting p-CY02 production compared with the other factors. The effects observed for NaF, SrCl_2_, NaHCO_3_, and Na_2_SiO_3_ on p-CY02 production were negative, and the effects observed for C_6_H_5_FeO_7_, NaCl, Na_2_SO_4_, CaCl_2_, KCl, KBr, and Na_2_HPO_4_ were positive, all with *p* values < 0.05. These seven compounds with positive effects and three compounds with nonsignificant effects for the production of p-CY02 were used in the second Plackett–Burman design.

To identify the ten components (C_6_H_5_FeO_7_, NaCl, MgCl_2_, Na_2_SO_4_, CaCl_2_, KCl, KBr, H_3_BO_3_, NH_4_NO_3_, and Na_2_HPO_4_) that have the greatest effect on p-CY02 production, the ten nutrient components selected through the preliminary Plackett–Burman design were retested using the Plackett–Burman design. The ten selected nutrient components were prepared at two concentrations (-1, +1) (Table S4). The design table and p-CY02 production data are shown in Table S5. The polynomial model describing the correlation between the ten components and the predicted p-RosPo-2 production is presented as follows:


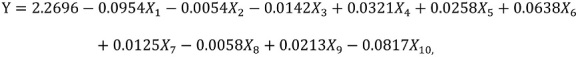


where Y is the predicted response (p-CY02 production) and X_1_–X_10_ are the coded values of C_6_H_5_FeO_7_, NaCl, MgCl_2_, Na_2_SO_4_, CaCl_2_, KCl, KBr, H_3_BO_3_, NH_4_NO_3_, and Na_2_HPO_4_, respectively.

Consequently, Table S6 shows the effect, *t* statistics, and *p* value for each nutrient component. From the regression analysis, KCl, Na_2_SO_4_, and CaCl_2_ were found to have positive effects (0.1275, 0.0642 and 0.0517); however, Na_2_HPO_4_ and C_6_H_5_FeO_7_ had negative effects (-0.1633 and -0.1908). A factor significant at the 95% level (*p* < 0.05) was considered to have a significant effect on p-CY02 production. The remaining compounds (NH_4_NO_3_, KBr, NaCl, H_3_BO_3_, and MgCl_2_) were not significantly different (*p* < 0.05). In conclusion, compounds (KCl, Na_2_SO_4_, and CaCl_2_) that exhibited a positive effect and had a significant impact at the 95% level (*p* < 0.05) compared to other compounds were considered to have a significant effect on the production of p-CY02. Consequently, these compounds were ultimately selected and utilized for central composite design. The concentration of the remaining compounds was maintained at the concentrations in the MB medium.

### Central Composite Design

The three nutrient components (KCl, Na_2_SO_4_, and CaCl_2_) selected through the Plackett–Burman design were tested at five concentrations (1.68, +1, 0, -1, and -1.68) (Table 1). The design table and p-CY02 production are shown in Table 2. The polynomial model describing the correlation between the three components and the predicted p-CY02 production is presented as follows:


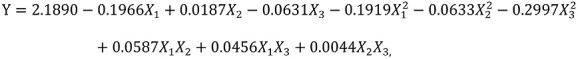


where Y is the predicted response (p-CY02 production) and X_1_ – X_3_ are the coded values of KCl, Na_2_SO_4_ and CaCl_2_, respectively.

The statistical significance of the second-order polynomial equation for the experimental data was evaluated by the *F* value and ANOVA (analysis of variance), which showed that the model was statistically significant at the 95%confidence level (*p* < 0.05), as shown in Table 3. An R^2^ value of 98.03% and an adjusted R^2^ value of 97.44% indicated good agreement between the experimental and predicted results for p-CY02 production in the present study. The model *F* value was 165.73, implying that the model was significant. The *p* value denotes the importance of each coefficient, helping in understanding the interaction between each independent variable [50]. In this study, the most significant sources of this model were X_1_, X_3_, X_1_^2^, X_2_^2^, X_3_^2^, X_1_X_2_ and X_1_X_3_ (*p* < 0.05), and *p* values greater than 0.10 indicated that the model sources were not significant (Table 3).

**Fig. 4:**
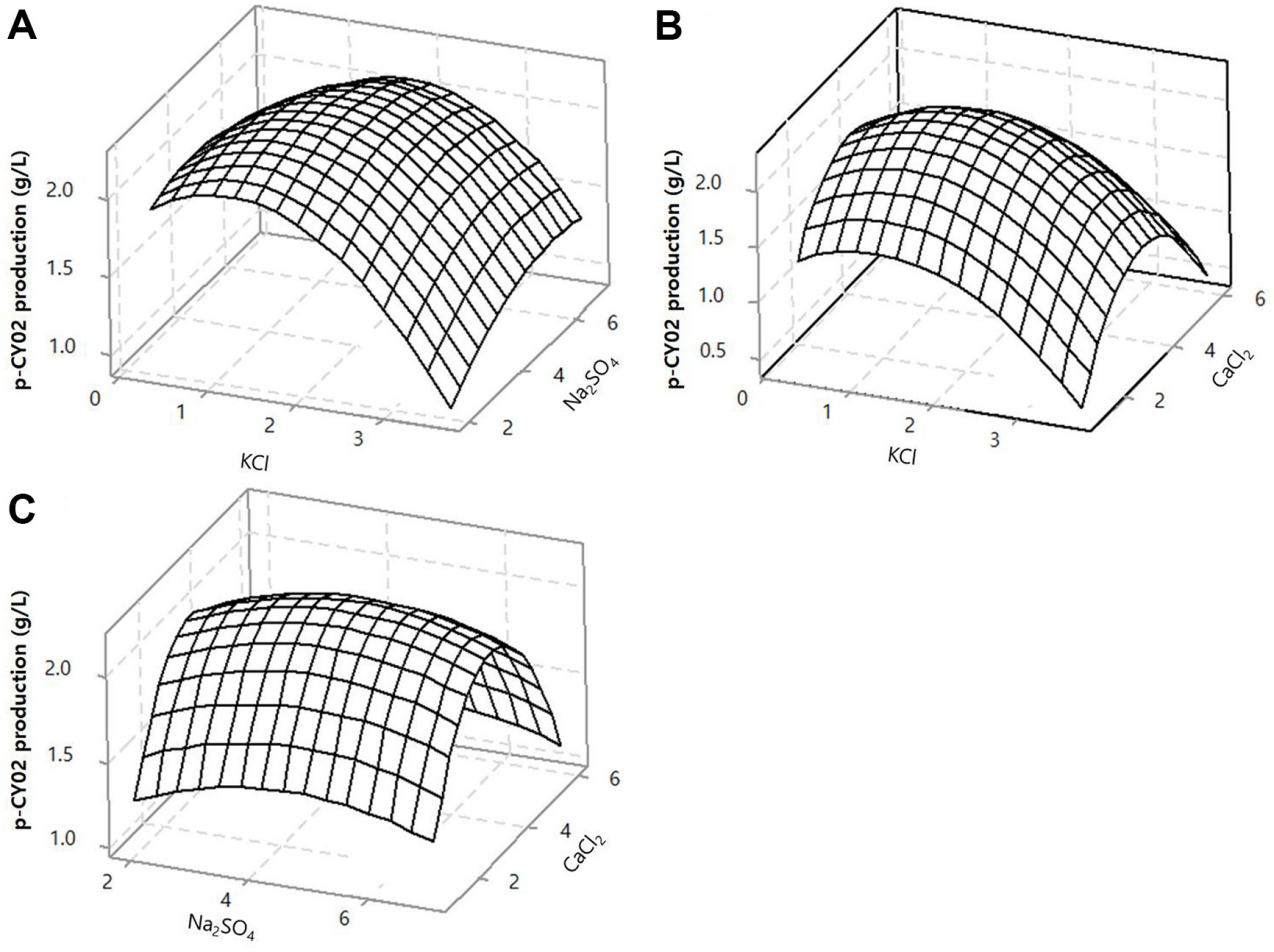
Three-dimensional response surface plot for the activity of (**A**) KCl in combination with Na_2_SO_4_; (**B**) KCl in combination with CaCl_2_; (**C**) Na_2_SO_4_ in combination with CaCl_2_ for p-CY02 production (g/l).

The main effect and interaction effects of the three factors KCl, Na_2_SO_4_, and CaCl_2_ at different concentrations are shown in Fig. 4. From this three-dimensional response surface plot, the optimal concentrations of KCl, Na_2_SO_4_ and CaCl_2_ were determined to be 1.43940, 4.32163, and 3.27066 g/l, respectively.

To confirm the results of the central composite design, *Pseudoalteromonas* sp. RosPo-2 was cultivated in the optimized medium (15.0 g/l fructose, 20.0 g/l yeast extract, 0.1 g/l C_6_H_5_FeO_7_, 19.45 g/l NaCl, 5.9 g/l MgCl_2_·6H_2_O, 1.43940 g/l KCl, 4.32163 g/l Na_2_SO_4_, 3.27066 g/l CaCl_2_·2H_2_O, 0.08 g/l KBr, 0.022 g/l H_3_BO_3_, 0.0016 g/l NH_4_NO_3_, 0.008 g/l Na_2_HPO_4_). The mean concentration of the produced p-CY02 was 2.21 ± 0.025 g/l, which was in good agreement with the predicted value (2.24639 g/l). The yield of p-CY02 was 1.64-fold greater than that of the MBG medium (1.35 ± 0.120 g/l).

## Discussion

Bacteria of the genus *Pseudoalteromonas*, known for producing EPS, are particularly abundant in Antarctic sea ice and seawater. Several studies have reported on the production yield of Arctic or Antarctic bacterial EPS. *Pseudoalteromonas* sp. MER144 was investigated under varying culture conditions, including temperature, pH, carbon source, and NaCl concentration, resulting in EPS production ranging from 43 mg/l to 318 mg/l [51]. *Pseudoalteromonas* sp. strain SM20310, isolated from the Arctic Sea, was selected from 13 strains due to its EPS production, which ranged from 50 mg/l to 576 mg/l. This EPS exhibited a cryoprotective effect and could enhance high-salinity tolerance [17]. *Pseudoalteromonas* sp. S8-8 from Antarctic sediment was studied to explore its industrial potential. The highest EPS production, reaching 163 mg/l, was obtained during growth in varying temperature, pH, carbon source, and NaCl concentration [52]. In this study, the RosPo-1 strain produced the highest EPS, but RosPo-2 strain was selected for its viscosity and cryoprotective properties; however, it also showed more EPS production (1.35 ± 0.120 g/l) compared to other *Pseudoalteromonas* strains from previous studies.

In one of these studies, EPS production by *Pseudoalteromonas* CAM025 was 30-fold greater at 2°C and 10°C than at 20°C [53]. Additionally, the chemical analysis of the EPS revealed a higher uronic acid ratio in samples produced at 2°C and 10°C compared to those at 20°C [54], while the neutral sugar content remained relatively unchanged. Meanwhile, another study showed that *Pseudoalteromonas* sp. S8-8 exhibited improved EPS production at 15°C compared to 4°C [52]. Furthermore, *Pseudoalteromonas* sp. SM20310 demonstrated the highest EPS production when cultured at 15°C, compared to cultures at 5°C and 30°C [17]. Referring to the results of these studies, the optimal culture temperature for p-CY02 production was established at 15°C. One of the key roles of EPSs is to safeguard surrounding cells in extreme environments [18, 55]. This effect is achieved through an increase in EPS viscosity due to hydrogen bonding between numerous hydrophilic side chains at low temperatures, thereby inhibiting ice crystal formation [15]. Consequently, the structural and physicochemical properties of EPSs are influenced by the length of the polymer chain [18]. The molecular weights of EPSs produced by Antarctic-derived microbes were 5–50 times greater than the average molecular weight of EPSs produced by other marine-derived microbes and ranged between 1 and 3 × 10^5^ Da [56]. For this reason, EPS-producing strains were isolated from the Ross Sea, including RosPo-1 and RosPo-2, which exhibited high viscosity among the strains collected and studied (Fig. 1B). We measured the cryoprotective effects of the final selected p-CY02 on HaCaT cells during freezing and thawing (Fig. 2A), and 0.8% (w/v) p-CY02 exhibited the maximum survival cell rate. DMSO, which is a permeating CPA, can reduce ice crystal formation, stabilize cell membranes, and scavenge oxygen free radicals during the freezing process. It can also however cause osmotic damage and toxicity due to the high concentration of DMSO [57-60]. Due to this toxicity, many attempts to lower the concentration have been undertaken [3, 61]. In previous research, the addition of EPSs was employed to measure the cryoprotective activity, as it helps reduce the required concentration of penetrating CPA and mitigates toxicity [35, 62].

Similarly, compared with the results of other reports, a cryoprotective activity test using a combination of p-CY02 and DMSO showed improved activity (Fig. 2B) [61-63]. For D-allose, the antifreeze activity was measured using 10% (w/v) D-allose while maintaining the concentration of the conventional CPA [61]; in the case of hydroxyethyl starch [64], a successful reduction in the concentration of DMSO was achieved using 5% (w/v) HES and 90% fetal calf serum (FCS) [62]. For the EPS of *Pseudomonas* sp. ID1, the applied concentration of EPS was 0.1% (w/v), while the concentration of the conventional CPA was maintained to measure cryoprotective activity [63]. In the present study, the concentration of p-CY02 was lower than that of HES, and p-CY02 clearly had a more pronounced effect on reducing DMSO than did D-allose, HES, and EPS from *Pseudomonas* sp. ID1. The final selected strain, RosPo-2 strain, was classified as a *Pseudoalteromonas* sp. based on the 16S rRNA gene sequence (Fig. 3). *Pseudoalteromonas* belongs to the class *Gammaproteobacteria* [65] and is the most abundant genus in the Arctic and Antarctic [66-68 as cited in Mancuso *et al*. 53].

Microbial EPSs have advantages in industrial applications due to their easy purification process and high production efficiency, which in combination address certain issues associated with plant- and animal-derived polysaccharides [21, 23]. Therefore, we employed RSM to increase the production of p-CY02, which exhibits cryoprotective activity. The production of p-CY02 in the optimized medium was 1.64-fold greater than that in the MBG medium. In this study, KCl, CaCl_2_ and Na_2_SO_4_, which have a positive effect on p-CY02 production (Table S6), also exhibit effects on increased EPS production in different microorganisms [69-71]. Potassium ion (KCl) is considered to contribute to the increase in EPS production because it serves as an intracellular signaling molecule, activating and/or inducing enzymes and transport systems to enable the cell to adapt to increased osmotic pressure [72]. Calcium ion (CaCl_2_) shows an effect on the regulation of EPS synthesis genes, leading to changes in mono-saccharide composition and increased production [73]. In previous studies, sulfur (Na_2_SO_4_) has been observed to induce stress responses in the fungus *Antrodia cinnamomea*, resulting in increased polysaccharide production [71]. Exposure of *Antrodia cinnamomea* to Na_2_SO_4_ led to elevated polysaccharide production as a protective response to osmotic stress [74]. However, it is noted that the relationship between Na_2_SO_4_ and polysaccharide production can be complex and context-dependent. Further research is necessary to fully understand the mechanisms underlying this relationship in various fungal species and environmental conditions. According to Suresh *et al*. [75], the yield and quality of microbial EPSs are strongly affected by nutritional and environmental conditions, and an increase in polymer production can be achieved by manipulating culture conditions. These results suggest that p-CY02 can be developed as a new, nonpermeating cryoprotective agent.

## Supplemental Materials

Supplementary data for this paper are available on-line only at http://jmb.or.kr.



## Figures and Tables

**Table 1:** The nutrient components and test levels for the central composite design.

Variable	Medium component	Ranges (g/l) and levels				
		1.68	1	0	-1	-1.68
X_1_	KCl	3.68179	3	2	1	0.31821
X_2_	Na_2_SO_4_	7.02269	6	4.5	3	1.97731
X_3_	CaCl_2_	6.02269	5	3.5	2	0.97731

**Table 2:** Central composite design matrix of nutrient components, along with predicted and observed values for p-CY02 production.

Trial No.	Variables^a^, Levels^b^			p-CY02	
	X_1_	X_2_	X_3_	Predicted value (g/l)	Production (g/l)
1	-1	-1	-1	1.984	2.003 ± 0.032
2	1	-1	-1	1.382	1.337 ± 0.011
3	-1	1	-1	1.895	1.833 ± 0.025
4	1	1	-1	1.528	1.553 ± 0.039
5	-1	-1	1	1.758	1.675 ± 0.028
6	1	-1	1	1.338	1.343 ± 0.004
7	-1	1	1	1.686	1.673 ± 0.032
8	1	1	1	1.502	1.425 ± 0.021
9	-1.68	0	0	1.977	2.033 ± 0.025
10	1.68	0	0	1.316	1.343 ± 0.039
11	0	-1.68	0	1.979	2.013 ± 0.039
12	0	1.68	0	2.041	2.090 ± 0.000
13	0	0	-1.68	1.447	1.453 ± 0.011
14	0	0	1.68	1.235	1.307 ± 0.011
15	0	0	0	2.189	2.118 ± 0.004
16	0	0	0	2.189	2.178 ± 0.004
17	0	0	0	2.189	2.225 ± 0.007
18	0	0	0	2.189	2.173 ± 0.004
19	0	0	0	2.189	2.232 ± 0.004
20	0	0	0	2.189	2.195 ± 0.035

^a^X_1_, KCl; X_2_, Na_2_SO_4_; X_3_, CaCl_2_

^b^+1, high concentration of variable; -1, low concentration of variable; 0, intermediate concentration of variable; -1.68 and +1.68, axial points.

**Table 3:** Analysis of variance of the central composite design for p-CY02 production.

Source	Sum of squares	df	Mean square	*F* Value	*p* Value
Model	462814	9	0.51424	165.73	0.000
X_1_	1.0561	1	1.0561	340.36	0.000
X_2_	0.00955	1	0.00955	3.08	0.090
X_3_	0.10888	1	0.10888	35.09	0.000
X_1_^2^	1.06124	1	1.06124	342.02	0.000
X_2_^2^	0.11542	1	0.11542	37.20	0.000
X_3_^2^	2.58917	1	2.58917	834.4	0.000
X_1_X_2_	0.05522	1	0.05522	17.80	0.000
X_1_X_3_	0.03331	1	0.03331	10.73	0.003
X_2_X_3_	0.00031	1	0.00031	0.10	0.756
Cor Total	4.72122	39			

R^2^ = 98.03%; R^2^ (adjust) = 97.44%

df: degrees of freedom, Cor: total corrected total sum of squares.
