# Supplementary material for: Optimization of Culture Medium for the Production of an Exopolysaccharide (p-CY02) with Cryoprotective Activity by Pseudoalteromonas sp. RosPo-2 from the Antarctic Sea
Source: J Microbiol Biotechnol. 2024 Mar 19;34(5):1135–45. doi: 10.4014/jmb.2402.02037 (PMC11180913; doi:10.4014/jmb.2402.02037)
Supplement: Supplementary file 1 [file jmb-34-5-1135-supple.pdf]

J. Microbiol. Biotechnol. 2024. 34(5): 1135–1145
https://doi.org/10.4014/jmb.2402.02037

Supplementary Tables

Table S1. The nutrient components and test levels for the initial Plackett–Burman experiment (elimination).

Variable	Medium component	Ranges (g/L) and levels	
		1	-1
X_1	$C_6H_5FeO_7$	0.1	0
X_2	NaCl	19.45	0
X_3	$MgCl_2$	5.9	0
X_4	Na_2SO_4	3.24	0
X_5	$CaCl_2$	1.8	0
X_6	KCl	0.55	0
X_7	$NaHCO_3$	0.16	0
X_8	KBr	0.08	0
X_9	$SrCl_2$	0.034	0
X_{10}	H_3BO_3	0.022	0
X_{11}	Na_2SiO_3	0.004	0
X_{12}	NaF	0.0024	0
X_{13}	NH_4NO_3	0.0016	0
X_{14}	Na_2HPO_4	0.008	0

Table S2. Initial Plackett–Burman (elimination) experimental design matrix of nutrient components and observed p-CY02 production.

Trial	Variables[a], Levels[b]														p-CY02
No.	X_1	X_2	X_3	X_4	X_5	X_6	X_7	X_8	X_9	X_{10}	X_{11}	X_{12}	X_{13}	X_{14}	production (g/L)
1	1	-1	1	1	-1	-1	-1	-1	1	-1	1	-1	1	1	ND[c]
2	1	1	-1	1	1	-1	-1	-1	-1	1	-1	1	-1	1	2.780 ± 0.078
3	-1	1	1	-1	1	1	-1	-1	-1	-1	1	-1	1	-1	1.963 ± 0.088
4	-1	-1	1	1	-1	1	1	-1	-1	-1	-1	1	-1	1	ND
5	1	-1	-1	1	1	-1	1	1	-1	-1	-1	-1	1	-1	2.340 ± 0.007
6	1	1	-1	-1	1	1	-1	1	1	-1	-1	-1	-1	1	2.968 ± 0.053
7	1	1	1	-1	-1	1	1	-1	1	1	-1	-1	-1	-1	ND
8	1	1	1	1	-1	-1	1	1	-1	1	1	-1	-1	-1	ND
9	-1	1	1	1	1	-1	-1	1	1	-1	1	1	-1	-1	2.117 ± 0.032
10	1	-1	1	1	1	1	-1	-1	1	1	-1	1	1	-1	2.625 ± 0.113
11	-1	1	-1	1	1	1	1	-1	-1	1	1	-1	1	1	2.417 ± 0.060
12	1	-1	1	-1	1	1	1	1	-1	-1	1	1	-1	1	2.455 ± 0.014
13	-1	1	-1	1	-1	1	1	1	1	-1	-1	1	1	-1	ND
14	-1	-1	1	-1	1	-1	1	1	1	1	-1	-1	1	1	2.165 ± 0.071
15	-1	-1	-1	1	-1	1	-1	1	1	1	1	-1	-1	1	ND
16	-1	-1	-1	-1	1	-1	1	-1	1	1	1	1	-1	-1	ND
17	1	-1	-1	-1	-1	1	-1	1	-1	1	1	1	1	-1	ND
18	1	1	-1	-1	-1	-1	1	-1	1	-1	1	1	1	1	ND
19	-1	1	1	-1	-1	-1	-1	1	-1	1	-1	1	1	1	ND
20	-1	-1	-1	-1	-1	-1	-1	-1	-1	-1	-1	-1	-1	-1	ND
21	0	0	0	0	0	0	0	0	0	0	0	0	0	0	2.570 ± 0.134
22	0	0	0	0	0	0	0	0	0	0	0	0	0	0	2.743 ± 0.018

[a] X_1, $C_6H_5FeO_7$; X_2, NaCl; X_3, $MgCl_2$; X_4, Na_2SO_4; X_5, $CaCl_2$; X_6, KCl; X_7, $NaHCO_3$; X_8, KBr; X_9, $SrCl_2$; X_{10}, H_3BO_3; X_{11}, Na_2SiO_3; X_{12}, NaF; X_{13}, NH_4NO_3; X_{14}, Na_2HPO_4

[b] +, high concentration of variable; -, low concentration of variable; 0, intermediate concentration of variable. [c] not detected.

Table S3. Statistical analysis of medium components using the initial Plackett–Burman design (elimination).

Variable	Medium component	Effect	Standard error	*t*-Statistics	*p*-Value
X_1	$C_6H_5FeO_7$	0.4505	0.0455	4.95	0
X_2	NaCl	0.266	0.0455	2.92	0.007
X_3	$MgCl_2$	0.082	0.0455	0.9	0.376
X_4	Na_2SO_4	0.273	0.0455	3	0.006
X_5	$CaCl_2$	2.183	0.0455	23.99	0
X_6	KCl	0.3025	0.0455	3.32	0.003
X_7	$NaHCO_3$	-0.3075	0.0455	-3.38	0.002
X_8	KBr	0.226	0.0455	2.48	0.02
X_9	$SrCl_2$	-0.208	0.0455	-2.29	0.03
X_{10}	H_3BO_3	-0.1855	0.0455	-2.04	0.051
X_{11}	Na_2SiO_3	-0.3925	0.0455	-4.31	0
X_{12}	NaF	-0.1875	0.0455	-2.06	0.049
X_{13}	NH_4NO_3	0.119	0.0455	1.31	0.202
X_{14}	Na_2HPO_4	0.374	0.0455	4.11	0

Table S4. The selected nutrient components and test levels for the secondary Plackett–Burman experiment.

Variable	Medium component	Ranges (g/L) and levels	
		1	-1
X_1	$C_6H_5FeO_7$	0.2	0.05
X_2	NaCl	22	15
X_3	$MgCl_2$	8	3
X_4	Na_2SO_4	4.5	1
X_5	$CaCl_2$	4	1
X_6	KCl	1.5	0.4
X_7	KBr	0.3	0.01
X_8	H_3BO_3	0.04	0.01
X_9	NH_4NO_3	0.004	0.004
X_{10}	Na_2HPO_4	0.04	0.001

Table S5. Secondary Plackett–Burman experimental design matrix of selected nutrient components and observed p-CY02 production.

Trial	Variables[a], Levels[b]										p-CY02
No.	X_1	X_2	X_3	X_4	X_5	X_6	X_7	X_8	X_9	X_{10}	production (g/L)
1	+	-	+	-	-	-	+	+	+	-	2.142 ± 0.053
2	+	+	-	+	-	-	-	+	+	+	2.058 ± 0.025
3	-	+	+	-	+	-	-	-	+	+	2.198 ± 0.039
4	+	-	+	+	-	+	-	-	-	+	2.115 ± 0.092
5	+	+	-	+	+	-	+	-	-	-	2.245 ± 0.035
6	+	+	+	-	+	+	-	+	-	-	2.265 ± 0.014
7	-	+	+	+	-	+	+	-	+	-	2.548 ± 0.074
8	-	-	+	+	+	-	+	+	-	+	2.265 ± 0.035
9	-	-	-	+	+	+	-	+	+	-	2.580 ± 0.049
10	+	-	-	-	+	+	+	-	+	+	2.220 ± 0.078
11	-	+	-	-	-	+	+	+	-	+	2.273 ± 0.004
12	-	-	-	-	-	-	-	-	-	-	2.327 ± 0.067
13	0	0	0	0	0	0	0	0	0	0	2.050 ± 0.007
14	0	0	0	0	0	0	0	0	0	0	2.003 ± 0.025

[a] X_1, $C_6H_5FeO_7$; X_2, NaCl; X_3, $MgCl_2$; X_4, Na_2SO_4; X_5, $CaCl_2$; X_6, KCl; X_7, KBr; X_8, H_3BO_3; X_9, NH_4NO_3; X_{10}, Na_2HPO_4
[b] +, high concentration of variable; -, low concentration of variable; 0, intermediate concentration of variable.

Table S6. Statistical analysis of medium components using the Plackett–Burman design.

Variable	Medium component	Effect	Standard error	*t*-Statistics	*p*-Value
X_1	$C_6H_5FeO_7$	-0.1908	0.010015	-9.53	0
X_2	NaCl	-0.0108	0.010015	-0.54	0.597
X_3	$MgCl_2$	-0.0283	0.010015	-1.41	0.178
X_4	Na_2SO_4	0.0642	0.010015	3.2	0.006
X_5	$CaCl_2$	0.0517	0.010015	2.58	0.021
X_6	KCl	0.1275	0.010015	6.37	0
X_7	KBr	0.025	0.010015	1.25	0.231
X_8	H_3BO_3	-0.0117	0.010015	-0.58	0.569
X_9	NH_4NO_3	0.0425	0.010015	2.12	0.051
X_{10}	Na_2HPO_4	-0.1633	0.010015	-8.15	0
